# Developing Disposable EEG Cap for Infant Recordings at the Neonatal Intensive Care Unit

**DOI:** 10.3390/s22207869

**Published:** 2022-10-16

**Authors:** Amirreza Asayesh, Elina Ilen, Marjo Metsäranta, Sampsa Vanhatalo

**Affiliations:** 1 BABA Center, Pediatric Research Center, Department of Clinical Neurophysiology and Pediatrics, Children’s Hospital and HUS Imaging, Helsinki University Central Hospital, HUS, 00029 Helsinki, Finland; 2 Department of Design, Aalto University, 02150 Espoo, Finland; 3 School of Industrial, Aerospace and Audiovisual Engineering of Terrassa-ESEIAAT, Department of Materials Science and Engineering, Universitat Politècnica de Catalunya, BarcelonaTech, 08222 Terrassa, Spain; 4 Department of Physiology, University of Helsinki, 00014 Helsinki, Finland

**Keywords:** aEEG, NICU, SAH, HF

## Abstract

Long-term EEG monitoring in neonatal intensive care units (NICU) is challenged with finding solutions for setting up and maintaining a sufficient recording quality with limited technical experience. The current study evaluates different solutions for the skin–electrode interface and develops a disposable EEG cap for newborn infants. Several alternative materials for the skin–electrode interface were compared to the conventional gel and paste: conductive textiles (textured and woven), conductive Velcro, sponge, super absorbent hydrogel (SAH), and hydro fiber sheets (HF). The comparisons included the assessment of dehydration and recordings of signal quality (skin interphase impedance and powerline (50 Hz) noise) for selected materials. The test recordings were performed using snap electrodes integrated into a forearm sleeve or a forehead band along with skin–electrode interfaces to mimic an EEG cap with the aim of long-term biosignal recording on unprepared skin. In the hydration test, conductive textiles and Velcro performed poorly. While the SAH and HF remained sufficiently hydrated for over 24 h in an incubator-mimicking environment, the sponge material was dehydrated during the first 12 h. Additionally, the SAH was found to have a fragile structure and was electrically prone to artifacts after 12 h. In the electrical impedance and recording comparisons of muscle activity, the results for thick-layer HF were comparable to the conventional gel on unprepared skin. Moreover, the mechanical instability measured by 1–2 Hz and 1–20 Hz normalized relative power spectrum density was comparable with clinical EEG recordings using subdermal electrodes. The results together suggest that thick-layer HF at the skin–electrode interface is an effective candidate for a preparation-free, long-term recording, with many advantages, such as long-lasting recording quality, easy use, and compatibility with sensitive infant skin contact.

## 1. Introduction

Long-term electroencephalography (EEG) recording is a rapidly expanding routine in neonatal intensive care units (NICU) [1,2]. It is commonly performed by applying four recording electrodes on selected locations (central (C3–C4) or parietal (P3–P4) and frontal (F3–F4)) as well as a reference and ground on the infant’s scalp [3,4]. These are interpreted using either a raw signal or a compressed display called amplitude-integrated EEG (aEEG). Despite the wide use of aEEG monitoring, it is commonly acknowledged that hooking up the infant and maintaining a quality recording over longer times is a bottleneck for the routine use of EEG monitoring [5,6], especially in centers with less technical expertise in EEG recording.

Dry EEG electrodes have improved comfortability in recent years and reduced patient preparation time [7,8,9]. They work with the method of direct resistive coupling without any extra skin–electrode interface material but based on the skin surface’s moisture and sweat. Skin–electrode impedance for these electrodes is higher than the wet electrodes [10] as they cannot make complete contact with the skin surface resulting in a noisier sequence in the signal outputs [11,12]. Therefore, active shielding or active dry electrodes are preferred to detract from environmental noises [13,14]. In semi-dry electrodes, the skin–electrode interface materials are replaced with liquid electrolytes, and the recorded signal quality and impedance level are comparable with wet electrodes. The first generation of semi-dry electrodes based on polymers had some issues with releasing the conductive solution without any control, resulting in unstable signal quality [15]. After some improvements, they could control the amount of saline to release. However, they need more progress in manually inserting the saline [16,17]. Hydrogels were introduced to eliminate the cleaning step at the end of the recording [18,19]. Hydrogel’s issue refers to the more comprehensive solution absorbing time that does not make them ideal for high-density electrodes. Additionally, textile electrode studies and their applications in wearable solutions for biosignal recording are expanding [6,20,21,22].

Long-term neonatal brain monitoring implies recording starts at any time of day, and it may last from hours to days [23]. Setting up and maintaining long-term EEG monitoring is challenged by the skills needed for skin preparation, electrode placement, and the maintenance of contact issues [24,25]. EEG recordings in the NICU have commonly interfered with artifacts from muscle activity, body movements, or various technical noises in the NICU environment [26]. In addition, an infant’s skin is sensitive and requires special measures to avoid skin lesions, excluding many common techniques in adults [20,27,28]. The current practice with wet skin electrodes using gel or paste is challenged by the labor needed to maintain the quality recording [29,30], with an easy emergence of noise that deteriorates the EEG signal [31]. In addition, an infant’s scalp is sensitive to contact pressure, limiting the use of EEG caps that are commonplace in EEG recordings of older subjects or short-term newborn EEGs [30,32]. Finally, the biocompatibility of the materials for the skin–electrode interface may limit the list of options in the NICU context.

This study set out to define the requirements for an optimal neonatal EEG cap and find a potential, scalable solution for long-term EEG recordings in NICUs. The requirements were systematically analyzed for all components in the cap, then potential solutions were examined for a biocompatible skin–electrode interface and the alternatives for connecting electrodes to wires. Then, the recordings were carried out on adult subjects and finally a disposable newborn EEG cap was constructed for a small series of proof-of-concept recordings in the NICU environment. Our results suggest a working end-to-end solution that could be produced as a disposable neonatal EEG cap with easy application and maintenance.

## 2. Materials and Methods

**Figure 1:**
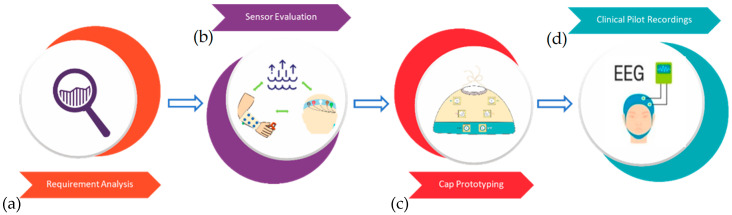
The study overview. (**a**) Analysis of requirements for a NICU-compatible EEG cap. (**b**) Technical comparison of different materials using an armband and headband. (**c**) Construction of a fully functional cap. (**d**) Proof-of-concept recordings in NICU.

An overview of the study rationale is presented in Figure 1. In brief, first the requirements were determined of an ideal NICU EEG cap, different technical solutions in lab recordings were tested, a fully functional neonatal cap was constructed, and, finally, proof-of-concept recordings were performed in the NICU.

### 2.1. Analysis of Requirements

Table 1 summarizes the requirements for an ideal NICU EEG cap for different parts of the whole cap solution, from the electrode–skin interface to user experience. Notably, requirements at all these levels must be met when designing a functional and clinically acceptable solution.

### 2.2. Sensor Evaluation

A metal sensor element is connected to the skin in the typical EEG solution using conductive media, recording gel, or paste [36]. A 10-mm-large Ag/AgCl disk element was chosen as the base solution in our cap design for connecting wires and the conductive media. Table 2 shows the different options evaluated as replacement of the conductive media, gel, or paste, which were used as conventional medical benchmarks.

#### 2.2.1. Dehydration Test

A dehydration test was performed to evaluate how well each material is able to retain water, a necessary characteristic of conductive material in long-term recordings [38]. This was conducted in open and closed spaces to compare with a typical NICU scenario with electrodes on the infant’s scalp in an open cot and a closed incubator. Different materials (Table 2) were compared to conventional gel and paste over 72-h periods, and the recordings were repeated three times. A 12 mm square shape from thin-layer HF and thick-layer HF, a Ø 12 mm SAH and sponge were taken as our dry materials, and 1.8 mL of paste, cream, electrogel, and Tensive gel; all materials were weighed before and after injecting 2% saline into them to assess their water absorption capacity. Then, the materials were left at room temperature on an open dish or in a plastic box (the latter was to replicate an NICU incubator setting). The materials were weighted at specific intervals for up to 72 h.

#### 2.2.2. Sensor Element

**Figure 2:**
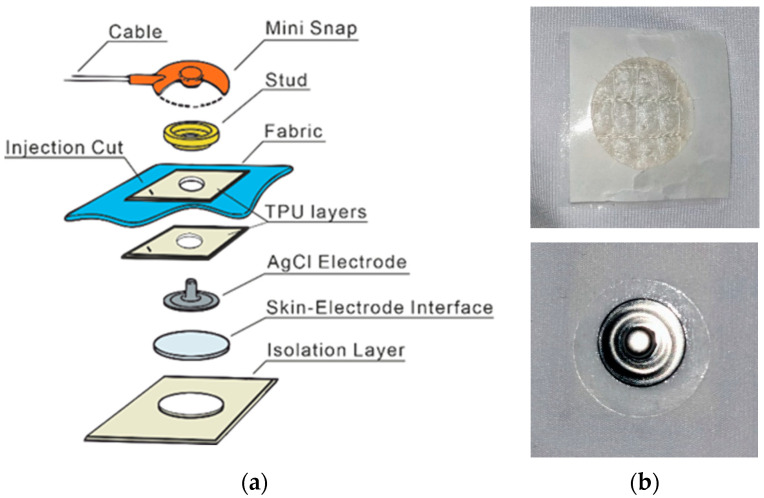
Schematic picture of the sensor element. (**a**) Components from the isolation layer to the stud are all pressed together to make up the entire sensor element in the cap. The isolation layer (TPU) faces the infant’s skin, the blue fabric depicts the cap textile, and the red button with cable is part of the bundle going to the amplifier. (**b**) Realistic sensor element prototype, including the snap electrode and skin–electrode interface sides.

The composition and realistic prototype of the sensor element on the sleeves, headbands, and cap are shown in Figure 2. An AgCl snap (appropriate with EEG/EMG cables (PD-h-2/L-80-905, Sorimex sp. z o.o. sp. k., Toruń, Poland) was pressed to the fabric over a TPU layer for all the electrode locations to protect the materials from quick dehydration and short circuit during or after the hydration. This layer has a 2 mm cut that allows easy access to the skin–electrode interface for hydration. Additionally, another TPU layer was applied to attach the skin–electrode interface material to the fabric. Because the SAH had a fragile structure after expansion and volume changes, instead of a TPU layer, a little pouch made of silver polyamide textile was made to hold it fixed in contact with the electrode. The adhesive Tensive gel was added to the reference and ground electrodes with a sticker surface to improve their skin adhesion during the long-term test recordings.

#### 2.2.3. Recordings Using a Forearm Sleeve and a Headband

Further tests using sleeves and headbands on an adult volunteer (A.A.) were performed on the best three alternatives from the previous studies (SPU, HF, and SAH). Here, long-term recordings were conducted on a real, though adult, skin surface. Recordings lasted for 24 h each and were repeated five times. For the first test, a forearm sleeve was equipped with four sensor elements (sponge (Ø 12 mm), commercial EMG/ECG sticker electrode, SAH (Ø 8 mm), and thick-layer HF (Ø 12 mm). The electrode diameters were nearly identical after hydration, although differences at this range do not affect the recording characteristics in a meaningful way [36]. Sticker electrodes (Kendall TM, Dublin, Ohio, USA) with added Tensive gel (Parker Laboratories, Inc., Fairfield, NJ, USA) were used as the reference and ground electrodes, separated from the other electrodes on the forearm sleeve. The reference electrode was placed on the opposite side of the elbow, and the ground electrode was placed on the palmar side of the wrist.

**Figure 3:**
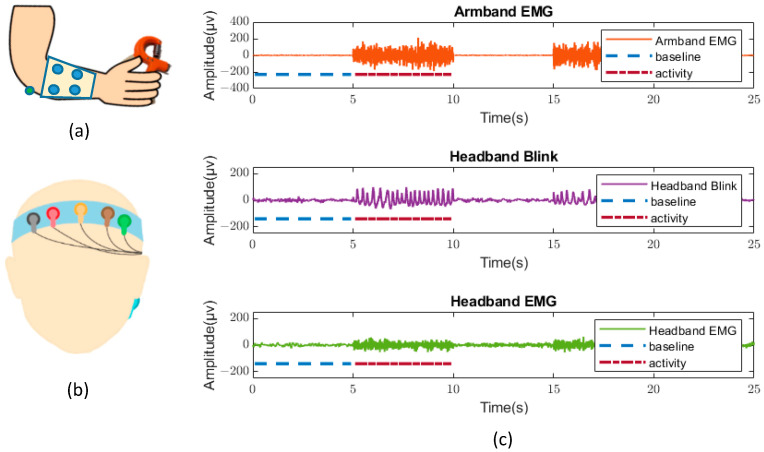
Schematic diagram of example recordings with the forearm sleeve and headband. (**a**) Forearm sleeve was used to record muscle activity vs. rest. (**b**) Headband was used to record blinks (eyelid closure) and frontal muscle activity (wrinkling of the forehead). (**c**) Sample recordings of the armband sleeve and the headband.

As represented in Figure 3, the headband had four sensor elements (two of each conductive material), two electrodes for reference, and ground placed in the middle of the forehead and beneath the ear, respectively. At the beginning of the experiment, the electrodes were hydrated with 2% saline water without any skin preparation and/or abrasion, and baseline measures were taken (see below).

During the 24-h recording, data were sampled every six hours for two minutes with a Bittium Brain Status Wireless EEG Amplifier (Bittium Corporation, Bothell, WA, USA) and associated software at a sampling rate of 250 Hz. In addition to measuring the electrode–skin impedance levels, the signal quality was also measured. High electrode–skin impedances can lead to signal distortion [31]. For clinical applications, electrode–scalp impedances exceeding 40 kΩ cause no significant attenuation in standard EEG frequency bands [29]. Because variations in the electrode–skin interface primarily cause motion artifacts, measuring the electrode–skin impedance is essential for determining the quality of an EEG signal in real-world applications [39].

This setup was coupled with four-channel EMG/EEG snap electrodes in a bipolar mode and analyzed using MATLAB 2021b (The MathWorks, Inc., Natick, MA, USA). A fourth-order bandpass filter was applied to the recorded data with low and high cutoff frequencies of 1 and 45 Hz, respectively. Testing the solutions of skin interphase with EEG signals is logistically demanding in the longer time spans used in our present study. Therefore, EMG recordings were used as a substitute for testing the skin interphase solutions to compare the electrode recordings on the armband and headband.

There were five trials with five seconds of baseline and five seconds of muscle activity per recording of two minutes. The subject was sitting at rest during the recording. Muscle activity in the forearm study was elicited by pressing a 3 kg chamber, whereas the headband activity was stimulated by blinking and EMG muscle tension that was recorded separately.

#### 2.2.4. Signal Analysis

The relative change in muscle activity vs. baseline was calculated to assess how well the given electrode materials can measure a clinically relevant biosignal. Though muscle activity is not fully equal to EEG activity, it has partly comparable frequency content and allows easier experimentation. The amount of muscle activation was computed as the Root Mean Square (RMS) median. In addition, the median RMS of the 50 Hz powerline component was estimated to calculate a proxy for the level of external noise that commonly interferes with the NICU EEG recordings [40]; this was computed as the difference between the raw signal and the signal after notch filtering.

#### 2.2.5. Cap Fabrication and Prototyping

The EEG cap was designed to be disposable because our interviews across several countries indicated that the re-use of patient contact devices of this kind might be prohibited in the NICUs in many countries. However, the cables connecting caps to the amplifiers can be made reusable. These requirements considerably impact the cap design within the constraints of suitable sales price targets (<100 EUR/USD): The cap needs to be made of relatively cheap raw materials and easy to manufacture with limited human labor. In addition, the textile products should allow automated cutting, i.e., laser cutting of pieces, and traditional sewing should be replaced by heat bonding with adhesive films in assembly.

**Figure 4:**
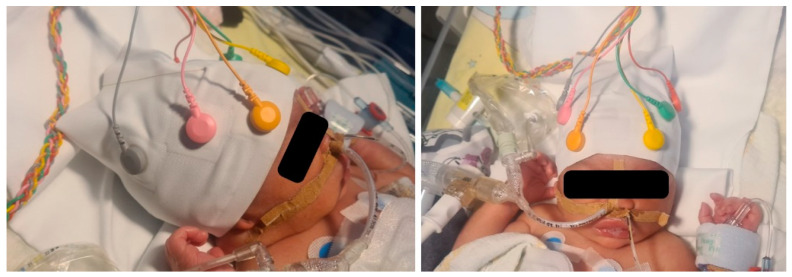
The EEG cap prototype with electrode connections.

An EEG cap was designed to house the required six electrode elements of clinical aEEG monitoring (4 active electrodes, reference, and ground) and allowed access to the mid-frontal area for possible ultrasound examination during the EEG recordings. After several iterations, many medical manufacturers commonly use the overall structure of a tube-shaped core as the holder for nasal tubes. Its shape is familiar to clinical nurses and appears to form an even, infant-friendly pressure on the desired scalp locations. In brief, the EEG cap consists of 6 textile-integrated sensors with medical-grade stainless steel snap buttons for connecting to the amplifier leads. The prototyped EEG electrode-cap system with electrode wire connections is presented in Figure 4 (see also above). Each electrode layer is heat bonded with waterproof TPU adhesive film to form the sensor element. TPU film is laminated on textile fabric and removes air gaps, generating a waterproof material. To avoid sensor shortcuts during the measurement, TPU layers were added on both sides of the cap fabric, which is fast-drying polyester, to minimize electrical conduction, i.e., shortcuts. An essential part of cap design is to reduce movement artifacts that arise from both moving the infant during care procedures and regular breathing when the head is lying on a pillow (a.k.a. breathing artifact). This is achieved by making the cap shape fit the infant’s head with some tolerable tension and adding height to the electrode–skin interface component (see above) to buffer movements. In our solution, the cap tension comes from the elasticity of the fabric and its 3D-engineered shape.

Moisture needed for the conduction at the skin–electrode interface comes from drops of saline (0.5–1.5 mL) injected into the skin–electrode interphase layer. Its evaporation is minimized using TPU film at different layers above the skin. Notably, rehydration of the sensor is possible with a blunt syringe from above using only water, and a little cut can be left near the snap button to make it possible to rehydrate during long-term recordings as needed. Outside the electrode element, the fabric is stretchable and permeable to water to add comfort and breathing over longer recording times. A silicone rim is added at selected locations around the head to avoid sliding the cap upwards during the longer recordings. These factors together help cap adjustment in infants with different head sizes and shapes; However, smaller cap sizes will be needed for at least studying preterm infants.

The following optimization was aimed in the manufacturing steps: The cap shape allows minimal fabric waste. Construction steps are minimized by using heat bonding lamination. First, the waterproof TPU layers are laminated on both sides of the fabric surface, followed by pressing snap buttons through the lamination. Then the sensor material is placed with TPU adhesive on top of it.

#### 2.2.6. Clinical Pilot Recording

Proof-of-concept recordings were performed from six newborn infants who underwent EEG recordings for their clinical indications. After the clinical EEG study, the EEG cap was placed on the subjects for 90–540 min. The subjects’ ages were 37–42 weeks (mean 41.8 weeks). Here, only caps with thick-layer HF elements were used that were hydrated with about 1 mL of tap water before applying the cap to the infant. Notably, no skin preparation was performed at the start of the recording.

The length of EEG recordings varied (1.5 h to 9 h) due to clinical situation, i.e., a convenience sample of EEG data were obtained. Six hours of data were selected from four subjects for the data analysis. The data were first read into MATLAB software and analyzed in bipolar derivations (F3–F4, P3–P4, F3–P3, and F4–P4). Analyses were averaged from three 10-s epochs at each 1 h. A 50 Hz notch filtering and a 6th order Butterworth bandpass filter (low-pass filter cutoff frequency: 35 Hz, high-pass filter cutoff frequency: 1 Hz) were applied. Then, the total relative power at the 1–2 Hz frequency range was computed. This was selected to estimate the amount of movement artifact; the results were benchmarked to a subdermal recording dataset. This EEG dataset was extracted from a previously published cohort [41] where infants were monitored for EEG activity due to clinical suspicion of stroke-related seizures. A detailed description of the clinical backgrounds of the ten infants included in this paper (#1,2,10–13,18,20,22,23) is presented in Supplementary Table S1 of [41]. Average 1–2 Hz activity in 24 h, one-hour epochs, which was used to assess how much the cap recording differs from the standard clinical recordings performed with subdermal needles in our NICU.

#### 2.2.7. Statistical Analysis

Statistical significance throughout the study was computed using Friedman’s and Dunn–Bonferroni’s post hoc test for multi-variate and Wilcoxon signed-rank for pairwise tests, employing a significance level of α = 0.05.

## 3. Results

In this section, the skin–electrode interface materials were first compared through the dehydration test; then their impedance, signal quality, and stability were compared in the armband and headband test. Then, the clinical proof-of-concept recording was performed by comparing the EEG cap recording by the prototyped cap with a cohort of archived EEG recordings using subdermal recordings.

### 3.1. Dehydration Test

**Figure 5:**
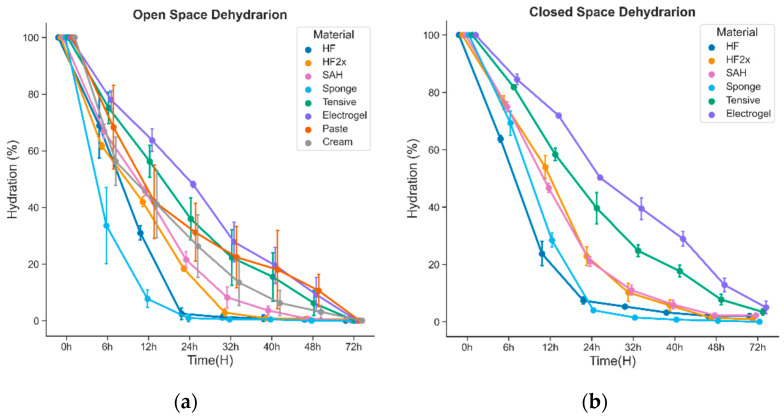
Results of dehydration tests for different types of conductive materials are shown separately for (**a**) the open space and (**b**) the closed space situation. The results are shown as loss of water, i.e., drop of weight as a function of time. The error bars (SEM) depict the results from *n* = 3 experiments.

Figure 5a presents the dehydration rate for all conductive medium materials tested: thin-layer and thick-layer HF, SAH, sponge, Tensive, electrogel, paste, and cream samples. Weights were compared between the start (time 0 h) and 72 h to estimate the amount of water that could evaporate from the product at room temperature and humidity. This allowed the relative loss of water over time to be assessed, as shown in Figure 5. The sponge was most rapid in dehydrating, followed by the thin-layer HF, while the other materials showed longer dehydration. The conventional electrode gels maintained longer dehydration than the other materials, though the difference was less robust and called for further testing on the forearm and headband settings (below).

### 3.2. Impedance Test

**Figure 6:**
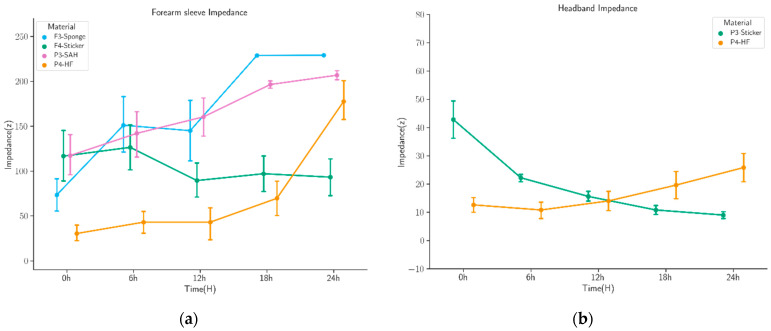
Impedance mean values of skin–electrode interface materials on the electrode–scalp impedance by measuring the 10 Hz impedance at the armband and headband. (**a**) The electrode–scalp impedance of the sponge, sticker, SAH, and HF overall measurements on the forearm sleeve. (**b**) The electrode–scalp impedance of the sticker and HF overall measurements on the headband.

Figure 6 shows changes in impedance in the forearm band (Figure 6a) and headband (Figure 6b) over the 24 h observation period. In Figure 6a, thick-layer HF has the lowest initial impedance (30 ± 9 kΩ) compared to other materials. The impedance score for the sponge dramatically increases after 12 h of recording because of abrupt evaporation. The SAH has high impedance values (over 100 kΩ) and fluctuations for the entire recording time. The standard sticker electrode starts with higher impedance values (116 ± 28 kΩ) but reaches more stable and lower values over time.

Regarding the impedance scores of stickers and thick-layer HF on the headband in Figure 6b, the initial impedance score for the HF (12 ± 3 kΩ) is significantly lower than the sticker (43 ± 7 kΩ). In contrast, the impedance level of the sticker reduces after 24 h of recording (9 ± 1 kΩ). Despite the increasing scores, the final impedance for the HF is (26 ± 11 kΩ).

In both tests, the thick-layer HF impedance increases steadily over time as well as exhibiting a low initial value.

### 3.3. Forearm and Headband Recordings

**Figure 7:**
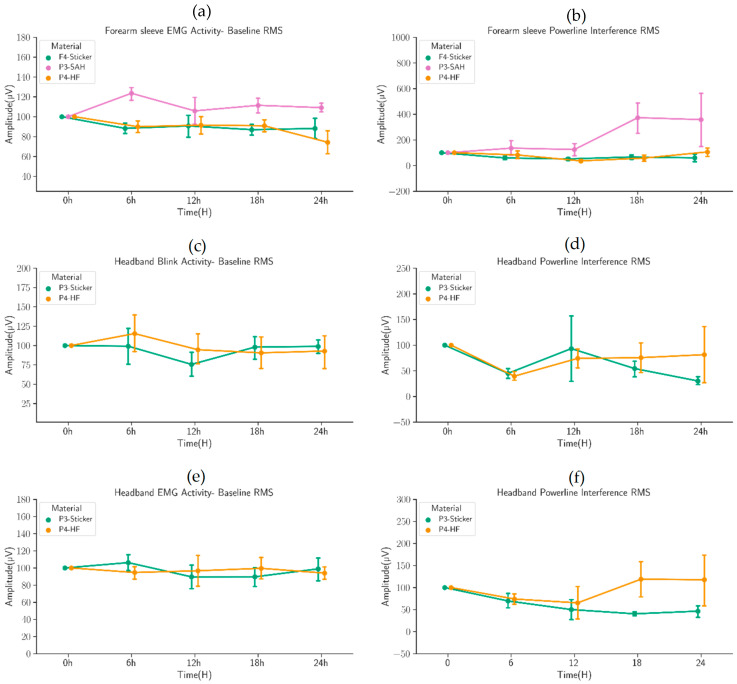
Comparison of contact medium in measuring activations using forearm sleeve and headband. The values are expressed as a percentage of the baseline activation. Note the general stability of the measures over 24 h recordings, suggesting that the contact media are comparable in biosignal recordings. (**a**) Forearm EMG activity-baseline RMS of Sticker electrode, SAH and HF. (**b**) Forearm EMG powerline interference RMS of Sticker electrode, SAH and HF. (**c**) Headband blink Activity-Baseline RMS of Sticker electrode, and HF. (**d**) Headband blink powerline interference RMS of Sticker electrode, and HF. (**e**) Headband EMG Activity-Baseline RMS of Sticker electrode, and HF. (**f**) Headband EMG powerline interference RMS of Sticker electrode, and HF.

Figure 7 summarizes the changes over time in the activation measures using the sticker (the conventional solution), SAH, and thick-layer HF as a conductive medium. The distribution of RMS values is comparable to the baseline shown in Figure 7a (Friedman’s test and Dunn–Bonferroni’s post hoc test; *n* = 25, *p* > 0.05). There is a significant increase in RMS with the SAH material over the recording shown in Figure 7b, and SAH turned out to be too fragile to continue with testing on headbands. Therefore, the headband was only tested with thick-layer HF.

Taken together, there was no significant change from the baseline activation, and the results were essentially comparable between the sticker and thick-layer HF contacts. Only powerline interference showed a wider variability with thick-layer HF than stickers, but the median values were comparable to the baseline. Therefore, thick-layer HF was considered to be a good enough alternative to sticker electrodes in recordings of muscle activation or blink-related biopotentials.

### 3.4. Clinical Proof-of-Concept Recordings

**Figure 8:**
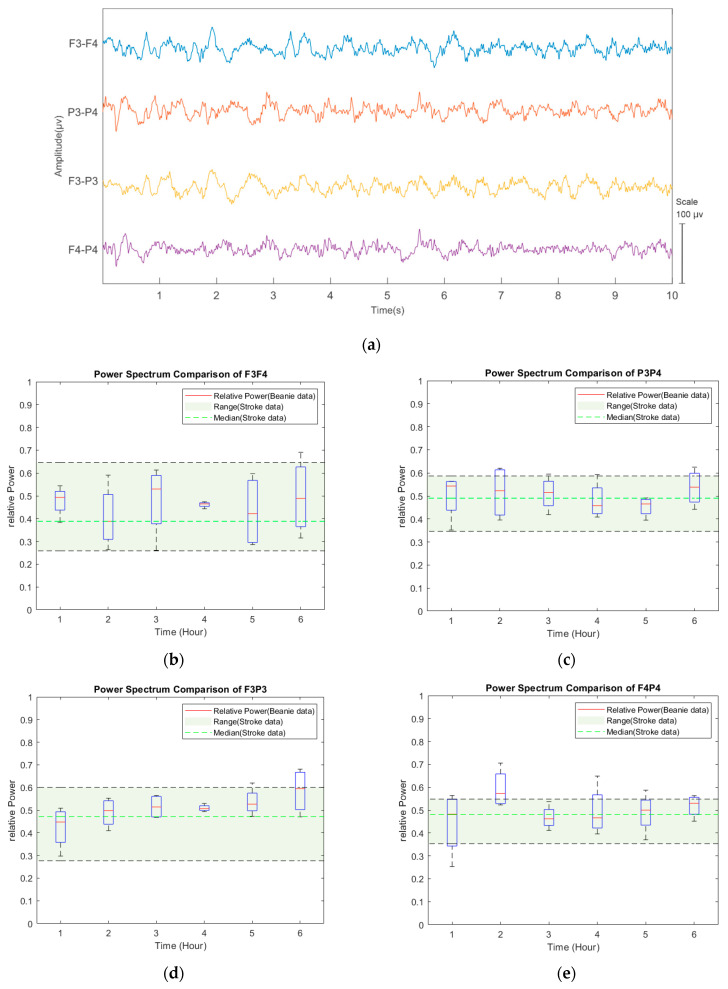

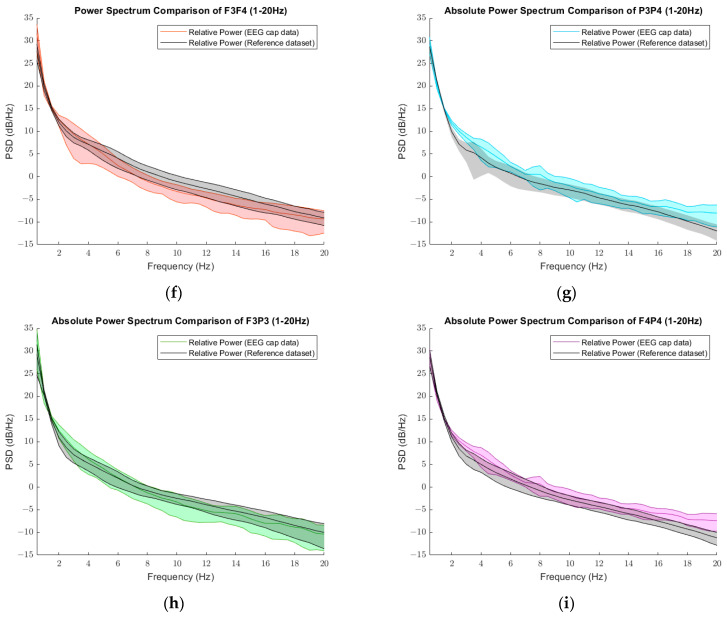
An example 10-s epoch of the raw EEG signals newborn infant with the novel EEG cap (**a**). Changes of 1–2 Hz relative power in different signal derivations and the corresponding measure are taken from a reference dataset (green zone). Asterisks denote time points with significant (*p* < 0.05) differences between datasets (**b**–**e**). Comparison of the relative power of 1–20 Hz between the prototype cap and the historical dataset recorded with subdermal needle electrodes (**f**–**i**).

Figure 8 shows an example EEG signal and relative power comparisons of the EEG cap recording quality over six hours compared to reference recordings obtained with subdermal EEG needles. The green zone in Figure 8b–e indicates the +/–2 SD range of the reference recording. The cap recording measures were comparable to the reference data for 6 h.

During the recording series, the cap design was slightly modified; we improved mechanical stability with additional silicone strip contacts between the cap fabric and skin at the temples and forearm area. This qualitative comparison showed that creating sufficient downward pressure on the electrodes is challenging when the cap does not include a chin strap. Adding a silicone lining to the edge of the cap created enough friction around the temples and forehead. In addition, the cap needed to be fitted low enough over the forehead (just over the eyebrows) and the occipital bone (just below the inion) to keep it in place better. Removing the cap is easy by only pushing it up from the ribs because the thick-layer HF does not stick to the skin surface. Moreover, most movement artifacts were related to changing the position of the electrode wires, which easily pulled the cap with electrode elements; this effect was only seen when the infant was moved, while the signals were stable during breathing movements when sleeping.

## 4. Discussion

The current study investigates alternatives for the design of neonatal EEG caps for NICU use. Our work shows that it is possible to construct a disposable cap for newborn EEG recording that provides sufficient signal quality without additional skin preparation and by using only added water for hydration at the skin interface. A thick-layer HF is shown to be suitable for replacing gel at the skin–electrode interface, allowing an EEG cap to be built that may even be disposable by design. The novelty of this study is to identify a solution for a cap structure that allows production as a disposable cap while also providing technically easy recording with minimal preparation and maintenance needed over longer-term aEEG monitoring in newborn infants. In contrast to previous approaches to aEEG recording, our concept includes a simple disposable setup that does not require any skin preparation, can be activated by tap water, and could be used in long-term recordings. This work does not challenge or change any prior knowledge on biosignal recordings; however, a series of previously experienced bottlenecks related to newborn EEG recordings were addressed. This study investigates the performance of multiple skin–electrode interface materials by comparing dehydration time, impedance, signal quality, and their general performance to conventional gels and pastes.

The dehydration test for long-term stability comparison and analysis for skin–electrode interface materials follows similar trends to the previous studies for dehydration and the loss of weight of the SAH material, which is around 20% hydration after 18 h [42]. Another critical factor in the stability is the structural change in the material after dehydration. For instance, the SAH material is fragile and has rigid corners after dehydration, which could harm the skin surface. The closed space dehydration experiment is admittedly not an exact replication of an infant incubator. However, it likely provides a close enough and feasible mimic of the NICU situation to compare the skin–electrode interface materials’ dehydration rates for long-term recordings.

Following the impedance and signal quality for different materials on the forearm and headband tests, to overcome the stability issues in long-term tests, Tensive gel was added to sticker electrodes to increase stability in the long-term recording. Although no skin preparation and extra pressure were carried out, the impedance range for the thick-layer HF was (12.6–25.8 kΩ) on the headband and (3.4–177.6 kΩ) on the armband in the long-term recording tests, while the impedances ranges in similar situations were (10.3–38.4 kΩ) for semi-dry electrodes, and (57.8–540.0 kΩ) for the dry electrodes [43]. The prior literature has shown that the skin impedance in the forearm locations may be several times higher than the forehead skin, presumably due to a thicker epithelial layer and lower density of sweat glands [44].

The actual clinical test recording comparisons with subdermal aEEG recordings indicated that a sufficient and nearly comparable signal quality and long-lasting performance are possible with the needle and hydrated skin–electrode interfaces.

A bottleneck in further clinical testing of the current prototype design was identified in the bulky electrode wires, which exit the cap as a bundle and may cause significant movement artifacts during the infant’s head movement. The electrode contacts were found to be mechanically stable enough to provide stable recordings. Some challenges in creating sufficient downward pressure were identified at the parietal electrodes, which could be solved by adding a silicone lining to the cap edge. These technical details need to be solved for mass production. In addition, to resolve the current issues, a wireless electrode setup could be designed to increase the mechanical stability of the electrodes and replace the amplifier and electrode wires out of the incubator, which could be targeted for future studies. This study sets a clear example for designing and manufacturing a disposable NICU-compatible (a)EEG cap that is user-friendly, provides sufficient signal quality over a long time, gives high patient comfort, and allows preparation-free application on the infant.

## Figures and Tables

**Table 1:** Requirements for components of the EEG cap.

Component	Requirements
Skin–electrode interface	Mechanical/Electrical stability of the skin-contacting element: Flexible, spongy, and sufficient contact friction in order to support a stable connection during infant’s movements while minimizing sliding over the skin Avoid too high contact pressure to minimize skin impressions Water-absorbing to maintain electrical conductivity over long times Suitable for different hair types Biocompatible
Cap and Wiring	Fabric: Breathable Ergonomic and comfortable for long-term recordings Compatible with NICU regulations Biocompatible Shape: Multiple sizes and flexible to fit on infants with widely-ranging ages and head shapes [33,34,35] Sturdy enough to fix the electrodes at selected scalp locations and to keep a constant skin pressure on the electrodes Wiring from the cap to the amplifier: Mechanically easy and electrically stable electrode contact at the caps Bundled wires. Flexible connector solution (different amplifiers)
User Experience	Installation: Easy, intuitive, and quick installation Color-coded electrodes Disposable according to NICU regulations Preparation and Checkups: No need for electrode or skin preparation. Quick setup for good signal quality Minimal and easy maintenance during long-term recordings (up to several days)

**Table 2:** Name, pros, and cons of the skin-electrode interface materials.

Name	Shape	Material	Pros	Cons
Conductive Fleece	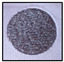	Silver coated polyamide	High conductivity Plain texture Thin material	Water repellent Tough fixation
Conductive Tape	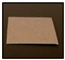	Silver coated tape	The adhesive on one side	Low conductivity Easy tearing
Conductive textured Fabric	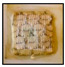	Polyester and silver-coated polyamide yarn	Quick hydration Soft and spongy structure	Hydration-dependent conductivity Quick dehydration
Sponge	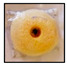	Silicone foam + TPU foam + TPU film, (Mepilex)	Spongy structure No sliding issues	Slow hydration
LoopVelcro	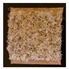	Silver-coated polyamide	High conductivity Less sliding on the skin surface Soft conductive lint	Water resistance Skin irritation Mechanical stability
Hook Velcro	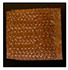	Silver-coated polyamide	High conductivity Stable connection and less sliding on the skin surface	Water-resistant Skin irritation Mechanical stability
SAH ^1^ Polymer	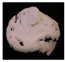	Hydrogel [18,19,37]	Slow dehydration time Spongy structure	Fragile Hard material before hydration
HF ^2^; Thin-Layer/Thick-Layer	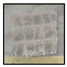	Natrium-carboxymethylcellulose Hydrogel	Spongy structure Quick hydration	Quick dehydration in a thin layer

^1^ Super absorbent hydrogel (SAH) polymer. ^2^ Hydro fiber.

## Data Availability

The datasets presented in this article are not readily available because they were provided by a third party (Helsinki University Hospital and University of Helsinki, Helsinki, Finland.)
